# A Pocket Medical Dictionary

**Published:** 1893-03

**Authors:** 


A POCKET MEDICAL DICTIONAY, giving the pronunciation and definition of about 12,000 of the principal words used in Medicine and the Collateral Sciences; by George M. Gould, A. M., M. D., author of "A New Medical Dictionary," Ophthalmic Surgeon to the Philadelphia Hospital; including very complete tables of the Arteries, Muscles, Nerves, Bacteria, Bacilli, Micrococci, Spirilli, and Flemometric Scales and a Dose List of Drugs and their preparations, in both the English and Matric Systems of Weights and Measures. Philadelphia: P. Blakiston, Son & Co. Price $1.00.

BASED as it is, upon his standard work, the author has given us a very complete and valuable little dictionary for every-day use, gotten up in convenient shape, and one which answers all practical purposes. C. E. J.

